# The geometric attention-aware network for lane detection in complex road scenes

**DOI:** 10.1371/journal.pone.0254521

**Published:** 2021-07-15

**Authors:** JianWu Long, ZeRan Yan, Lang Peng, Tong Li

**Affiliations:** 1 College of Computer Science and Engineering, Chongqing University of Technology, Chongqing, China; 2 NullMax (Shanghai) and Co. Ltd, Shanghai, China; Tongii University, CHINA

## Abstract

Lane detection in complex road scenes is still a challenging task due to poor lighting conditions, interference of irrelevant road markings or signs, etc. To solve the problem of lane detection in the various complex road scenes, we proposed a geometric attention-aware network (GAAN) for lane detection. The proposed GAAN adopted a multi-task branch architecture, and used the attention information propagation (AIP) module to perform communication between branches, then the geometric attention-aware (GAA) module was used to complete feature fusion. In order to verify the lane detection effect of the proposed model in this paper, the experiments were conducted on the CULane dataset, TuSimple dataset, and BDD100K dataset. The experimental results show that our method performs well compared with the current excellent lane line detection networks.

## Introduction

Lane detection is a basic but still challenging task [1–5] in perceptions of autonomous vehicle, which requires that algorithm can detect the lane lines from traffic scene image captured by car cameras. Some recent works have defined lane detection as a pixel-intensive prediction task [6–8]. Segmented lane lines are available for trajectory tracking control and positioning vehicles in autonomous driving, then detected lanes can be used to judge the status of other traffic participants. In addition, it is also a pivotal part of making highly precision maps and crashing prediction [9–11].

Recently, the most studies about lane detection have been seen as the semantic segmentation tasks [6–8, 12], but they severely rely on labels which are sparse and fixed-width as supervision signals of fully convolution network to classify foreground (lane line) or background pixel by pixel. Although some methods can segment lane lines accurately in some traffic conditions with good weather and wide views, the realistic driving scenes are often complicated and changeable. In traffic jam scenes, considerably blocked cars would cover the lane lines, which makes fully convolution network tends to predict discontinuous or fuzzy lane lines. Therefore, these situations bring great challenges to lane detection methods based on semantic segmentation.

Nowadays, there have been several solutions proposed in complex road scenes to improve lane detection accuracy. First, expanding the receptive field of fully convolution network to infer the characteristics of lane lines from "global perspective", such as ASPP of Deeplabv3 [13], and the backbone of fully convolutional networks is designed to be very deep for better understanding the target globally, such as ResNet [14] and DenseNet [15]. Second, Increasing the ability of messaging among neurons in the network to encode richer semantic context. Third, using a multi-task network [16] architecture to predict more lane lines characteristics and improve lane detection in adverse conditions. Fourth, training the model in high-quality and large-scale datasets annotate unclear or obscured lane lines artificially, which makes the network learns abundant features.

**Fig 1:**
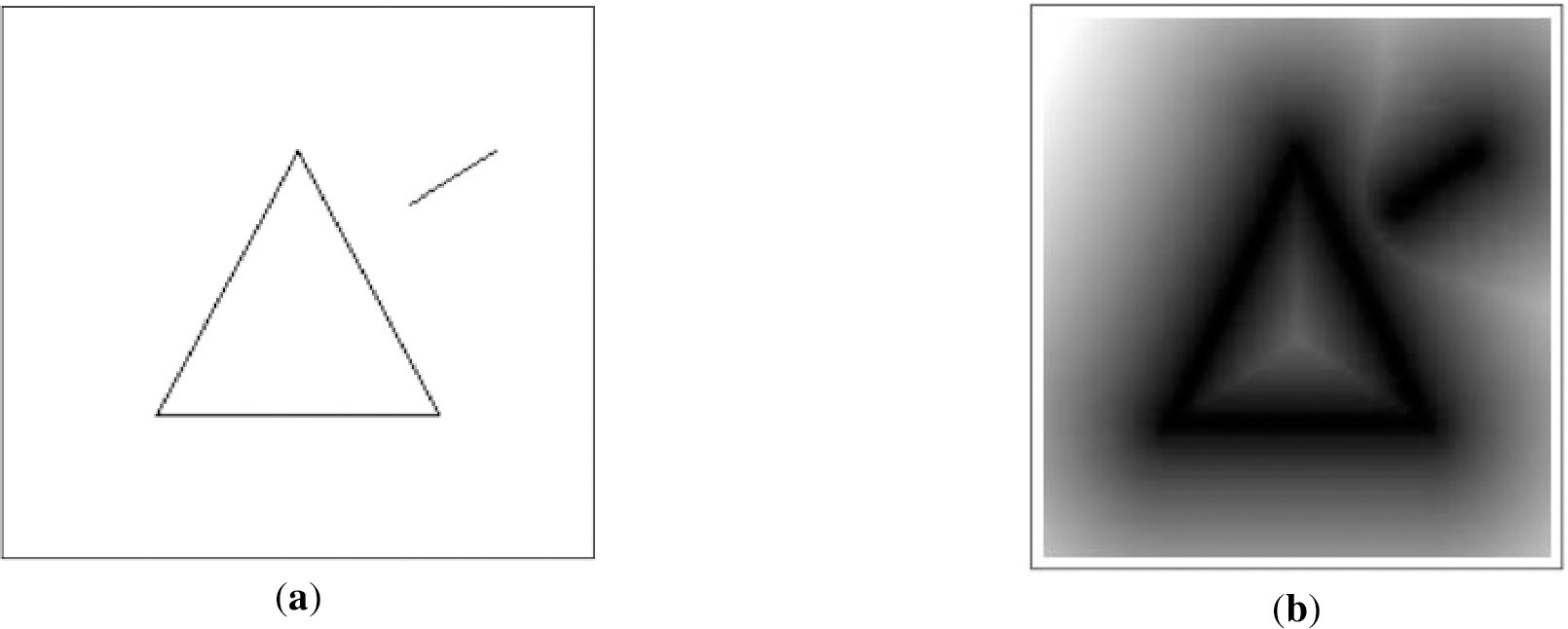
(**a**) One-hot mask; (**b**) Distance transform mask.

However, in complex road scenes, these lane detection methods above don’t perform very well in accuracy. Semantic segmentation methods based on fully convolution network merely generate black or white classification predictions for each pixel according to the one-hot mask. This kind of models are easy to generate fuzzy segmentation results on the target boundary, as well as, usually influenced by noises to cause misclassification. Thus, we introduce the distance transform [17] mask as shown in Fig 1B, which is a continuous representation and each pixel represents the minimum distance to a nearby line segment or boundary. Compared with the one-hot mask (Fig 1A) used for classification, the gradient is smoother when model performs back propagation by this method.

On the distance transform mask, Audebert et al. [16] introduced a cascaded multi-task loss based on distance transformation to improve the effect of boundary segmentation. Hayder et al. [18] used this mask instead of the one-hot mask to solve the problem of poor segmentation due to inaccurate object candidate frames. In [19], the authors transformed the regression prediction into a distance segmentation mask task, to focus more on pixels near boundaries and improve the segmentation results of target boundaries in satellite images. Although the above methods have introduced distance transformation to overcome the boundary leaky problem in semantic segmentation, they treat multi-task branches as independent tasks or simply fuse feature maps.

Therefore, we propose the GAAN to solve the problems mentioned above, which allows the model using the geometric distance information of the lane lines to guide segmentation and enhance the network’s understanding of the semantic context information. Concretely, GAAN adopts multi-task branches neural network architecture. The first branch, semantic segmentation, is to predict the lane lines. The second branch is geometric distance embedding, which is to predict the minimum Euclidean distance of boundary pixels from the center to the lane line in regression. In each branch, we use an architecture which can autonomously select required information for communication, and call it as AIP module.

Moreover, we design a GAA module on the tail of the two branches to obtain the features with geometric distance information, then we fuse the final high-dimensional lane line features, which leads to the result containing missing or wrong lane line features in the semantic segmentation branch can be repaired and predicted correctly. Finally, the different level semantic features in the encoder are fused by Skip Pyramid Fusion Up-sampling (SPFU) module, which restores the prediction of lane boundary pixels better.

## Materials and methods

### Geometric distance transform label

As described in the previous section, essentially most lane detection algorithms based on semantic segmentation are pixel-by-pixel classification tasks. However, if prediction between predicted pixel and real label has just slight deviation, the penalty cost of the loss function to the network is equal to wrong prediction. This "ignorance" is not fair. Hence the kind of hard classification method is not beneficial for the segmentation of the lane boundary in complex scenes. According to this, in this paper, it is recommended to predict the geometric distance transformed labels to improve the semantic segmentation effect of the lane line in complex scenes. The distance transformed label is a continuous representation that encodes each pixel on the lane line.

**Fig 2:**
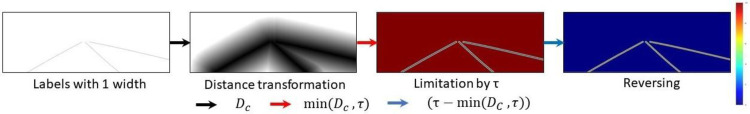
Process of obtaining geometric distance transformation mask.

Producing the masks is very simple and convenient, which only needs to be adjusted on the original lane line labels. Specifically, we demonstrate the process of generating geometric distance transformed label in Fig 2. Firstly, the one-hot labels in lane datasets are sampled by pixel coordinates of the central part in each lane line, and mark the sampled lane as the width 1 pixel line. Then we calculate the distance transformation based on one-hot labels and reveal transformation result in the second step, which illustrates the minimum Euclidean distance from each pixel to the nearby lane line. Moreover, we set a threshold *τ* to limit the range of the distance transformation area to eliminate the influence of invalid value in regression, and *τ* is related to the width of lane line in the label mask. However, the distance transformed label is a continuously increasing distance from the center of the lane line to the boundary, which adds redundant noise areas to the regression task. Finally, reversing the truncated distance mask, so that the geometric distance is continuously reduced from the center of the lane line to the boundary to 0, and the distance transformation mask d_*mask*_ can be formulated as following:

$$d_{mask}=(\tau -\min\left(\min\left(d_{p}\right),\tau \right))$$ (1)

where min(*d*_*p*_) is the minimum Euclidean distance from a certain pixel p to the nearby lane line, τ is truncated threshold. Hence, the distance mask transforms the lane mask from a line into the range area.

The geometric distance transformed mask described above has the following advantages over the one-hot label mask:

1. The lane line pixels on transformed label mask encodes the distance information to the boundary, which contributes to improve the segmentation of the boundary.
2. Compared with the category information in the one-hot label mask, each pixel in the distance transformed label mask has specific distance information. The accurate information may reduce the impact on redundant noise.

### The framework of geometric attention perception network

**Fig 3:**
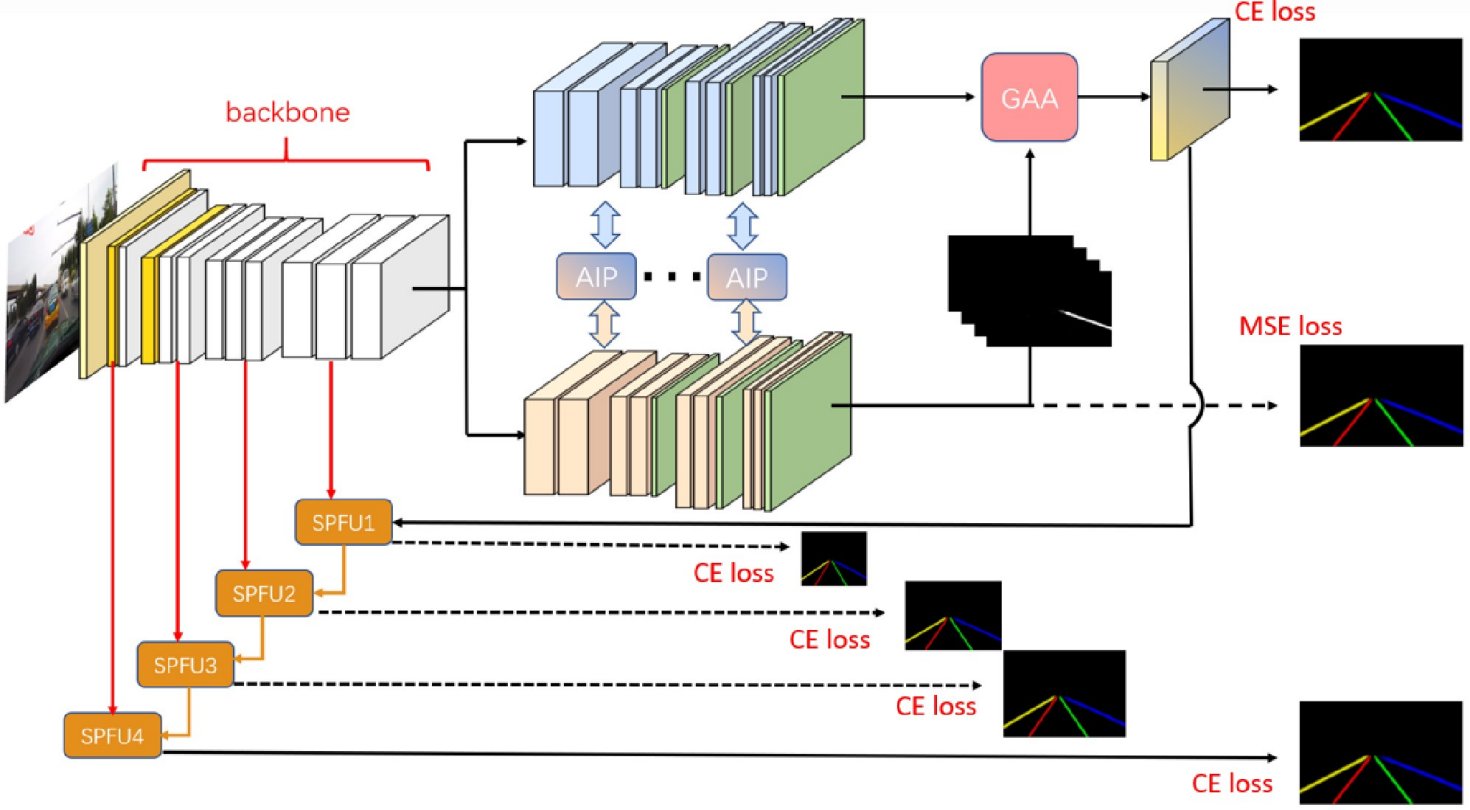
The GAAN.

In this section, an overview of end-to-end deep convolutional neural network designed to detect lane lines in complex road scenes is demonstrated. As shown in Fig 3 is the framework of GAAN consisted of 6 parts which include the backbone network, the semantic segmentation branch, the geometric distance embedding branch, the AIP module, the GAA module, and the SPFU module.

The backbone network maps the RGB images to the high-dimensional feature space and the two branches behind the backbone network reconstruct the geometric distance embedding and lane line semantic labels from shared high-dimensional features. The feature information communication between the two task branches is performed by AIP module. The module adaptively selects the feature information for fusion, and then GAA module combines distance embedding features and semantic features. In short, this module fuses the two branches of feature map, which include long distance information and contextual semantics respectively. Final step, every feature map in the backbone network are restore by SPFU module, which can combine with GAA module to gradually generate different resolution of the feature maps, so as to use the loss function for supervision during training.

In our backbone network architecture, ResNet is appropriately modified to enhance its expressed ability of lane features. As shown in Fig 3, we divide the backbone into 4 layers, where the yellow parts in layer 1 and 2 are down-sampling layer with 2 step size, which is benefit for keeping the spatial information in the feature map. Then layer 3 and 4 use atrous convolution (dilated convolutions) to capture a wider range of contextual semantic information.

### Attention information propagation module

The information sharing and information propagation play a significant role in the network with multi-task branches, while the sharing and propagating strategy between branches is difficult to manually adjust. Therefore, we introduce the AIP module to complete it, which selects weight on each channel and automatically selects different branches to output feature maps in a learnable way.

AIP module is located between the two up-sampling layers of the decoders. There are three AIP modules between the two branches. The lane feature information extracted by the backbone is not only propagated in the relative task branch, but also share information from the other task branch through AIP module which selects and fuses features from the current branch and the other branch.

**Fig 4:**
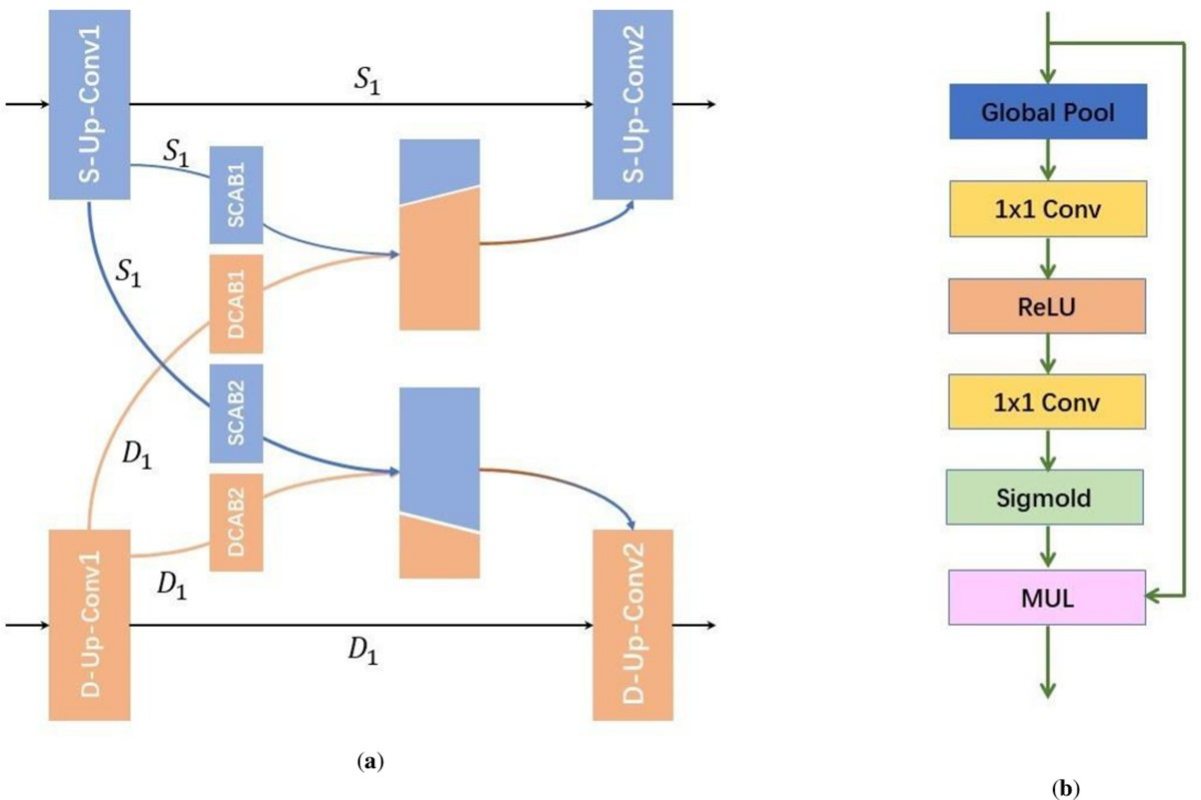
(**a**) AIP module; (**b**) Channel attention block.

Concretely, as shown in Fig 4A, we display the first AIP module as an example. The first layer in the semantic segmentation branch is the S-Up-Conv1 and the output feature map is named *S*_1_. The first layer in the geometric distance embedding branch is the D-Up-Conv1 and the output feature map is named *D*_1_. DCAB and SCAB are channel attention block of distance embedding branch and segmentation branch respectively. The propagation of the attention information can be defined as Eq 2, where *α*_1_ and *α*_2_ are the channel attention weight of feature map *S*_1_ respectively, *β*_1_ and *β*_2_ are the channel attention weight of feature map *D*_1_. The channel attention block (CAB) is shown in Fig 4B that we first calculate the global average pooling of the input features to obtain a feature vector containing global context information, then calculate 1x1 convolution and activation function for this feature vector. Besides we name shared information are ${AIPM}_{S_{2}}$ and ${AIPM}_{D_{2}}$, which will be sent to the subsequent layer.


$$\left\{ \begin{array}{l} {AIPM}_{S_{2}}=S_{1}+(\alpha_{1}S_{1}+\beta_{1}D_{1})\\ {AIPM}_{D_{2}}=D_{1}+(\alpha_{2}S_{1}+\beta_{2}D_{1}) \end{array}\right.$$ (2)

Although all AIP modules have the same structure, their parameters are irrelevant, which makes information propagation more flexible between stages of the multi-task network. Furthermore, *S*_1_ is identity mapping information to the next up-sampling layer, which ensures the propagation of the internal information of the branch and avoids the interruption of the propagation during the network training process. This residual-like idea is also conducive to the back propagation of the gradient.

### Geometric attention-aware module

The geometric distance embedding branch predicts the continuous distance from the lane line’s center to boundary by regression. This branch which extracts feature map with lane line geometry information to guide the results of semantic branch segmentation has higher tolerance than the semantic segmentation task that is pixel-by-pixel classification. Therefore, we introduce the GAA module locates in the end of the two task branches, which captures the context information between long-distance lane lines from the high-dimensional feature distance of geometric distance embedding. Information includes boundary distance context information, which is more beneficial to the segmentation of the entire lane line and boundary pixels.

The first step of GAA module is to decouple the input geometric distance embedded features to generate a spatial attention matrix, which simulates the spatial relationship between any two pixels in the feature map. The second step is to compute multiplication between the attention feature matrix and the semantic segmentation feature matrix. The third step is to compute an element-wise sum operation on the result of second step, and obtain the final information that reflects the long-range contextual geometric information.

**Fig 5:**
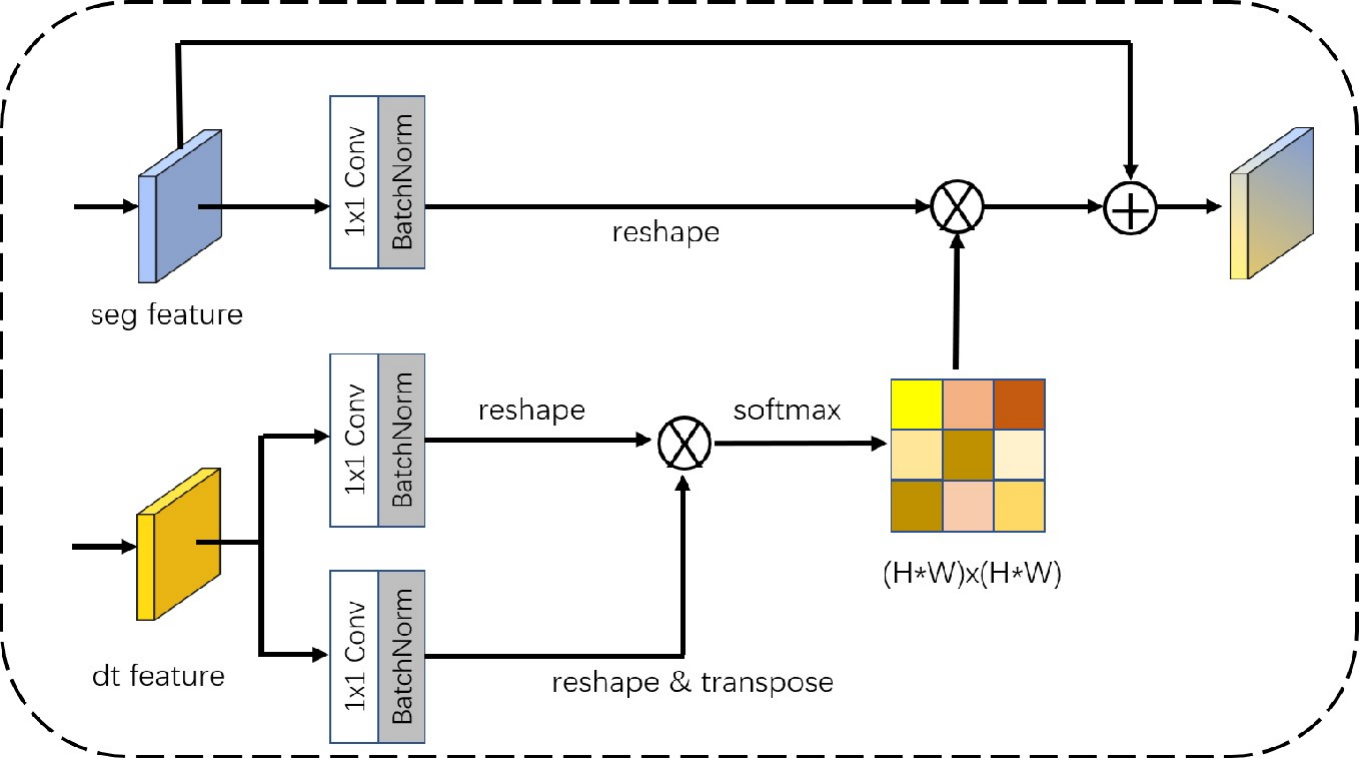
The GAA module.

The specific working process of this module is shown in Fig 5. Given the semantic segmentation branch output feature *A*∈ℝ^*C*×*H*×*W*^, the output feature of geometric distance embedding branch is decoupled through two 1x1 convolutional layers, and the shape of new features are *B*∈ℝ^*C*×*H*×*W*^ and *C*∈ℝ^*C*×*H*×*W*^, then we reshape features *B* and *C* to ℝ^*C*×*N*^, where *N* = *H*×*W* is the number of pixels. In addition, we perform a transpose operation on feature *C*, the result of transpose computes matrix multiplication on the reshaped features *B* and *C*. Finally, we use the SoftMax to calculate the spatial attention map *S*∈ℝ^*N*×*N*^, the calculation process is shown in Eq 3:

$$S_{ji}=\frac{e^{\left(B_{i}\times C_{j}\right)}}{\sum_{i=1}^{N}e^{\left(B_{i}\times C_{j}\right)}}$$ (3)

where *S*_*ji*_ measures the influence of the spatial position *i*^*th*^ on the position *j*^*th*^, and the more similar feature representation of the two positions contributes to their greater correlation. At the same time, the output of semantic segmentation branch is sent to the 1x1 convolution layer to generate a new feature map *D*∈ℝ^*C*×*H*×*W*^, and reshaped it to ℝ^*C*×*N*^. Then computing matrix multiplication between features *D* and *S*, and reshaped the result to ℝ^*C*×*H*×*W*^. Finally, the result and feature A compute element-wise sum to obtain GAA module’s output *E*∈ℝ^*C*×*H*×*W*^, for position *j*^*th*^ is shown in Eq 4.


$$E_{j}=\sum_{i=1}^{N}(S_{ji}\times D_{i})+A_{j}$$ (4)

It can be concluded from Eq 4 that each element on the feature map finally output by GAA module is the weighted sum of the geometric distance and the semantic segmentation feature map. Therefore, it has rich global context geometric features, and adaptively aggregates context information through the spatial attention, which improves the continuity of lane lines’ prediction.

### Skip Pyramid Fusion Up-sampling module

After the encoder and decoder, the image resolution is continuously changed, which would lead to lose detail information in the feature map. In our network, for solving this case, SPFU (Skip Pyramid Fusion Up-sampling) module is proposed to restore more lane line high-quality detail information in the final semantic feature map. As mentioned in the previous content, Fig 3, SPFU module uses the extracted image feature with different granularity levels through skip connection. Thus, we choose the feature maps of some middle layers in encoder.

**Fig 6:**
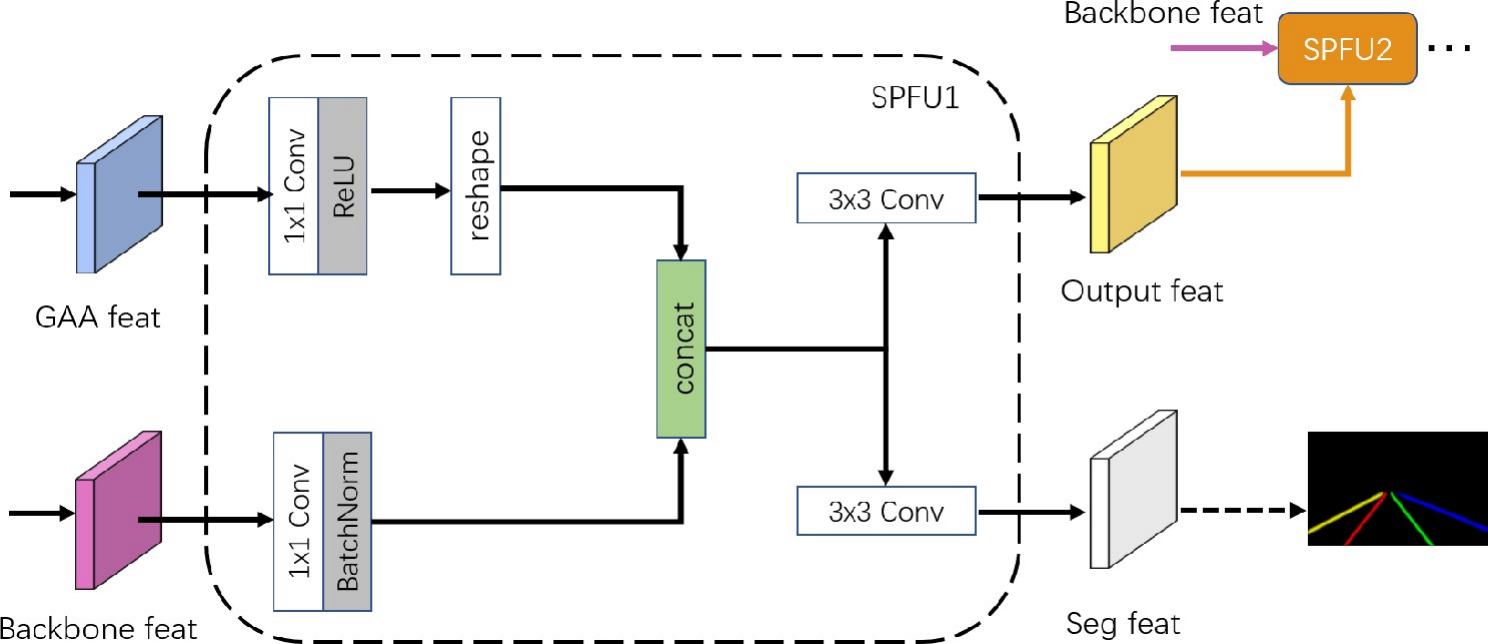
SPFU module.

We show the first SPFU module named SPFU1 as an example. As shown in Figs 3 and 6, the input of SPFU1 is the final feature map of GAA module and the backbone. After computing the 1x1 convolution and generating new feature maps, then we adjust the shape of feature maps so that they can be contacted. Finally, we compute two 3x3 convolutions separately, one convolutional result is to fuse features with the next backbone feature map for SPFU2, it is next-stage SPFU module, another convolutional result is to supervise the semantic segmentation loss function.

### Loss function

Most semantic segmentation methods use cross entropy to measure the difference between the prediction and ground truth. However, cross entropy loss is more suitable for natural images with complete and large objects, and the lane lines are very long and thin in the lane datasets, which contain a lot of background pixels that are not conducive to predicting targets. Therefore, it is necessary to use a weighted cross-entropy loss function to supervise the semantic segmentation branch training, because this loss can effectively control the influence of each category of pixels to the cross-entropy loss function by setting different weights. Its definition is shown in Eq 5:

$$L_{seg}(A,\hat{A})=-\frac{1}{N}\sum_{i=1}^{C}\omega_{i}\times {\hat{A}}_{i}\times log(\varphi (A_{i}))$$ (5)

where *A*∈ℝ^*H*×*W*^ is the final output calculated by semantic segmentation branch, *φ*(.) is the Softmax. After Softmax *φ*(.), the feature map *A* generates the lane line probability map. *N* is the total number of pixels in the feature map. *ω* is the loss contribution weight of each prediction category. Usually we set the background weight to 0.4 and the remaining lane line weights to 1 in the CULane dataset.

In the geometric distance embedding branch, we want to predict the continuous distance from the center line to the boundary for each lane line, which shows that it is not a classification task but a regression prediction. Therefore, we use the MSE (mean square error) in GAAN to measures the error between the geometric distance embedded branch prediction result and the real label, this process is shown in Eq 6:

$$L_{dt}(B,\hat{B})=\frac{1}{N}\sum_{i=1}^{C}{\left\|B_{i}-{\hat{B}}_{i}\right\|}^{2}$$ (6)

where *B*∈ℝ^*H*×*W*^ is the final output of geometric distance embedding branch, ${\hat{B}}_{i}$ is the geometric distance mask d_*mask*_.

To sum up, the total loss function is shown in Eq 7:

$$L_{total}=L_{seg}+L_{dt}+\alpha \sum_{k=1}^{k}L_{segk}+\beta L_{exist}$$ (7)

where $L_{seg}$ is the weighted cross-entropy loss function of the semantic segmentation branch, $L_{dt}$ is the mean square error loss function of the geometric distance embedding branch, $L_{segk}$ is the semantic segmentation auxiliary loss function, which is used to supervise the feature map output by the SKPFU module. $L_{exist}$ is a binary cross-entropy used to supervise the existence of lane lines, or it predicts whether lane lines exist in the image. *α* and *β* are hyperparameters.

## Results

### Datasets and evaluation

In order to verify the effectiveness of the GAAN in lane detection of complex road scenes, experiments were conducted on the TuSimple dataset, the CULane dataset, and the BDD100K dataset. Various detailed traffic scenarios are divided in order to evaluate the detection results in different scenarios.

**Fig 7:**
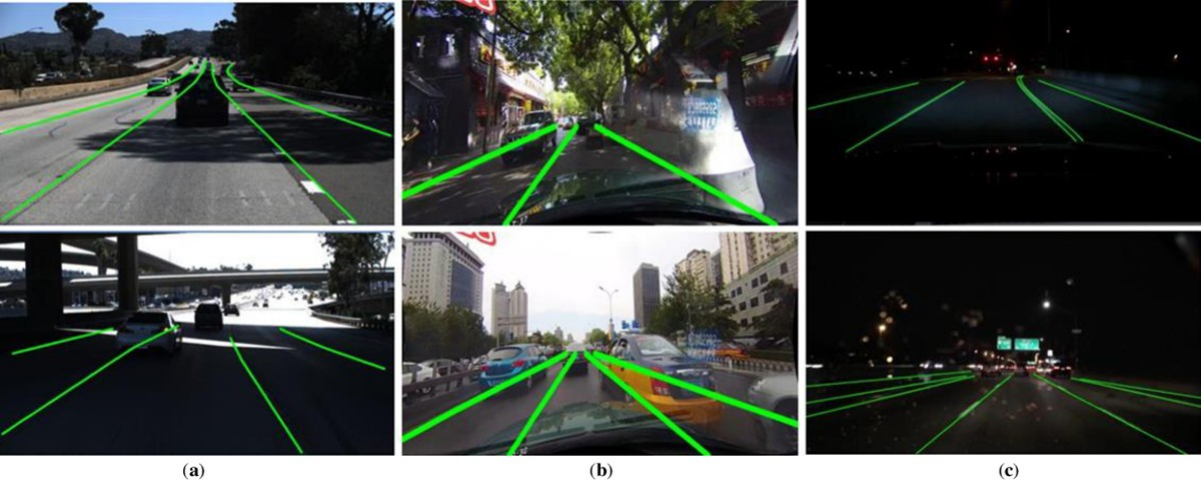
Examples of three datasets. (**a**) TuSimple dataset; (**b**) CuLane dataset; (**c**) BDD100K dataset.

In above three datasets, the TuSimple dataset focuses on highway scenes, the CULane dataset and the BDD100K dataset mainly focus on urban road scenes. The BDD100K dataset was originally used for lane instance segmentation, which contains rich types of lane line and annotates instance for the same type of lane lines. It can be seen from Fig 7 that the TuSimple dataset, the CULane dataset and the BDD100K dataset all contain different complex road scenes, where the green line is the labels of the lane line. In these examples, it is not difficult to find that characters of lane are unclear because trees’ shadow on the roadside and other crowded cars in traffic scenes leading serious obstruction to lane‘s character, as well as, lighting angle and insufficient lighting at night.

Table 1 is the detailed description of above three datasets containing complex road scenes. The second column is the total number of frames in per dataset, and the third, fourth, fifth columns are the number of images that are divided into training set, validation set, testing set in the extracted frames. Besides the seventh column is road type of dataset and the eighth column is the number of lane lines.

**Table 1:** Information about datasets.

Dataset	Frames	Training	Validation	Test	Resolution	Type	Lane
TuSimple	6408	3268	358	2782	1280×720	Highway	4、5
CULane	133235	88880	9675	34680	1640×590	Highway、City	4
BDD100K	80000	60000	10000	10000	1280×720	Highway、City	8

All experiments in this work were performed in the following environment: a workstation containing two NVIDIA GEFORCE RTX 2080Ti, each 2080Ti is 11GB, the operating system is Ubuntu 18.4, all experiments perform training and inference use the pytorch deep learning framework.

In our work, we respectively reshaped the images of TuSimple, CULane and BDD100K to 368×640, 288×800 and 360×640 as input size. When training the GAAN, we use SGD optimizer to train the model, the learning rate is set to 0.01, the learning rate update strategy is ploy, the learning rate attenuation coefficient is 0.9, the BatchSize is set to 12. TuSimple’s training iterations are set to 1800, CULane and BDD100K are set to 60K respectively. The hyperparameters α, β in the final loss function are set to 0.1 respectively.

To test the performance of our model, we used Accuracy, False Positive (FP), and False Negative (FN) on the TuSimple dataset as evaluation. The CULane dataset use F1-Measure, FP and The BDD100K dataset uses Accuracy, IoU as evaluation respectively. The calculation methods for these evaluation indicators are described in following:

$$FP=\frac{F_{pred}}{N_{pred}}$$ (8)

where *F*_*pred*_ is the number of lanes with incorrect predictions, and *N*_*pred*_ is the number of all predicted lanes.

$$FN=\frac{M_{pred}}{N_{gt}}$$ (9)

where *M*_*pred*_ is the number of wrong predicted lane lines, and *N*_*gt*_ is the number of ground truth.

$$Accuracy=\frac{\sum_{clip}C_{clip}}{\sum_{clip}S_{clip}}$$ (10)

where *C*_*clip*_ is the predicted lane pixels. *S*_*clip*_ represents the total effective lane line pixels.

$$Fmeasure=(1+\beta^{2})\frac{Precision*Recall}{\beta^{2}Precision+Recall}$$ (11)

where *β* = 1 and the *Precision*, *Recall* is shown in Eqs 12, 13 and 14 is *IoU*:

$$Precision=\frac{TP}{TP+FP}$$ (12)

$$Recall=\frac{TP}{TP+FN}$$ (13)

$$IoU=\frac{TP}{TP+FP+FN}$$ (14)

### Experiments

Table 2 shows the lane detection F1-Measure of the GAAN on the CULane testing set. Compared with the current other advanced lane detection algorithms on CULane dataset, we can find that the proposed method performed very well among seven different complex road scenes and the total testing set, where RD101-GAAN indicates that ResNet101 with deformable convolution [21] is used as the backbone network in the GAAN.

**Table 2:** F1-Measure of several methods on CULane dataset.

Scene Method	normal	crowd	hlight	shadow	noline
SCNN [8]	90.6	69.7	58.5	66.9	43.4
GCJ [9]	89.7	**76.5**	67.4	65.5	35.1
SAD [4]	90.7	70.0	59.9	67.0	43.5
RD101-GAAN	**93.2**	74.5	**68.1**	**73.3**	**47.6**

The reason why the GAAN can perform well in complex road scenes is that the geometric distance embedding branch contains the geometric information of the lane boundary, which can effectively guide the result of semantic segmentation through the GAA module. However, the F1-Measure of the GAAN in the crowded scene is lower than GCJ [9] in Tables 2 and 3, since GCJ designed a loss function about geometric relationship between driving area and lane lines for supervision. That is, the segmentation result of the driving area has a strong correlation with the lane lines, so that the lane lines can be inferred from the driving area. In addition, since there is no ground truth in the Crossroad scene, only the FP evaluation index is counted.

**Table 3:** F1-Measure of several methods on CULane dataset.

Scene Method	arrow	curve	crossroad	night	total
SCNN [8]	84.1	64.4	**1990**	66.1	71.6
GCJ [9]	82.2	63.2	-	68.7	73.1
SAD [4]	84.4	65.7	2052	66.3	71.8
RD101-GAAN	**87.4**	**70.9**	2126	**72.5**	**75.8**

In order to verify the effectiveness of the GAAN’s components, the ablation experiments were performed by gradually adding components after the backbone network ResNet-50. As shown in Tables 4 and 5, Only-Dt represents that there is only one distance embedded branch in the network, and its lane line detection result is worse than Only-Seg which only uses semantic segmentation branch. Seg-Dt represents that semantic segmentation branch and geometric distance embedding branch are simultaneously trained and predicted, which has better performance than when only using a single task branch. Later, we gradually add the AIP module, GAA module and SPFU module on the basis of the Seg-Dt. It can be seen that with the increase of components in the network, its F1-Measure has also gradually increased on each scene of the CULane dataset, which illustrates that each component plays a positive role in the performance of lane detection.

**Table 4:** Ablation experiments of GAAN with different modules.

Scene Model	normal	crowd	hlight	shadow	noline
Only-Dt	85.2	62.6	49.1	59.6	33.0
Only-Seg	88.5	66.2	57.3	65.4	37.9
Seg-Dt	89.4	67.3	58.5	66.0	38.7
Seg-Dt-AIPM	90.3	68.1	62.9	66.5	41.4
Seg-Dt-AIPM-GAA	91.0	69.5	63.4	67.6	43.3
Seg-Dt-AIPM-GAA-SPFU	**91.7**	**73.0**	**65.6**	**69.4**	**45.0**

**Table 5:** Ablation experiments of GAAN with different modules.

Scene Model	arrow	curve	crossroad	night	total
Only-Dt	79.5	53.3	**1405**	57.3	64.8
Only-Seg	82.1	61.1	1888	63.3	68.8
Seg-Dt	83.1	63.9	1728	64.8	69.3
Seg-Dt-AIPM	84.3	65.3	1815	65.0	70.4
Seg-Dt-AIPM-GAA	85.0	66.2	1492	66.3	71.6
Seg-Dt-AIPM-GAA-SPFU	**85.3**	**66.9**	1546	**68.2**	**72.9**

In the computer vision task based on deep learning, the feature expression ability of the encoder has a decisive influence on the extraction effect of the target feature by the entire neural network. Therefore, different encoders are explored for GAAN’s impact of detecting lanes. As shown in Tables 6 and 7, using ResNet-50, ResNet-101, and ResNet-DConv-101 as the backbone network for GAAN, where ResNet-DConv-101 is based on ResNet-101 network and the grid convolutional replaced by the deformable convolution. As the complexity of the backbone network increasing, the network can better extract lane line features, and the better results of lane detection in complex scenes.

**Table 6:** Ablation experiments of GAAN with different backbone.

Scene Model	normal	crowd	hlight	shadow	noline
R-50-GAAN	91.7	73.0	65.6	69.4	45.0
R-101-GAAN	92.5	73.7	66.2	72.0	46.3
RD-101-GAAN	**93.2**	**74.5**	**68.1**	**73.3**	**47.6**

**Table 7:** Ablation experiments of GAAN with different backbone.

Scene Model	arrow	curve	crossroad	night	total
R-50-GAAN	85.3	66.9	**1546**	68.2	72.9
R-101-GAAN	86.1	68.4	1674	70.7	74.2
RD-101-GAAN	**87.4**	**70.9**	2126	**72.5**	**75.8**

**Fig 8:**
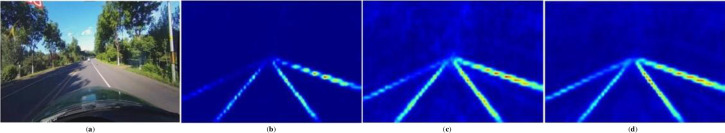
Heatmap of GAAN with different modules: (**a**) Input; (**b**) Seg; (**c**) GAA module; (**d**) SPFU.

In order to demonstrate the effect of each module in the ablation experiment on the lane line detection intuitively and clearly, we show a lane image on the left side of the road occluded by shadows and input to the trained GAAN with RetNet-50 backbone. The results are displayed as a heat map as shown in Fig 8. As we continue to add modules, the results can be seen in the heat maps from left to right, the entire model has improved the ability to recognize the lane line features, especially the leftmost blocked lane line has increasingly clear inference. Furthermore, the feature map output by the SPFU4 module fuses information from all levels of the encoder, and the lane line noise is significantly reduced in the output result.

**Fig 9:**
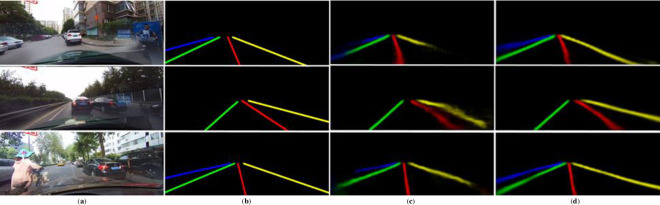
Comparison of GAAN and SCNN, (**a**) Input; (**b**) GT; (**c**) SCNN; (**d**) GAAN.

As shown in Fig 9, in order to qualitatively describe the ability of the GAAN to detect lane lines in complex road scenes, we select three results from the CULane testing set to illustrate our method’s advanced performance. In comparison, the GAAN performs better in detection which lanes are covered by the car on the left side than SCNN, in that the GAA module can capture the long-distance dependencies between pixels. In addition, the input images of row 2 and 3 are traffic scenes where crowded vehicles covered lane lines. The detection results of the GAAN are also better than SCNN’s, because our method suffers less redundant noise and the pixels of the same lane lines predicted are more consistent.

As shown in Table 8, we evaluated the GAAN on the TuSimple dataset and compared it with other networks that performed well on the dataset.

**Table 8:** Evaluation of GAAN in TuSimple dataset.

Method	Accuracy	FP	FN
LaneNet [5]	96.38	0.0780	0.0244
SCNN [8]	96.53	0.0617	0.0180
EL-GAN [20]	96.39	**0.0412**	0.0336
ENet-SAD [4]	96.64	0.0602	0.0205
RD-101-GAAN	**96.75**	0.0576	**0.0173**

The labels of BDD100K are different from the TuSimple and the CULane. BDD100K labels the lane lines that can be seen in the image, instead of focusing only on the 4 lane lines on the left and right sides of the current lane in the same direction. Thus the dataset contains the distribution of samples with different numbers of lane lines, which leads the lane detection results are greatly influenced. Therefore, the ability of the network model’s learning and generalization can be effectively verified on BDD100K dataset. As shown in Table 9, it illustrates the evaluation results of the GAAN on the BDD100K dataset.

**Table 9:** Evaluation of GAAN in BDD100K.

Method	Accuracy	IoU
ResNet-101 [11]	34.45	15.02
SCNN [8]	35.79	15.84
ENet-SAD [4]	36.56	16.02
RD-101-GAAN	**37.68**	**16.75**

**Fig 10:**
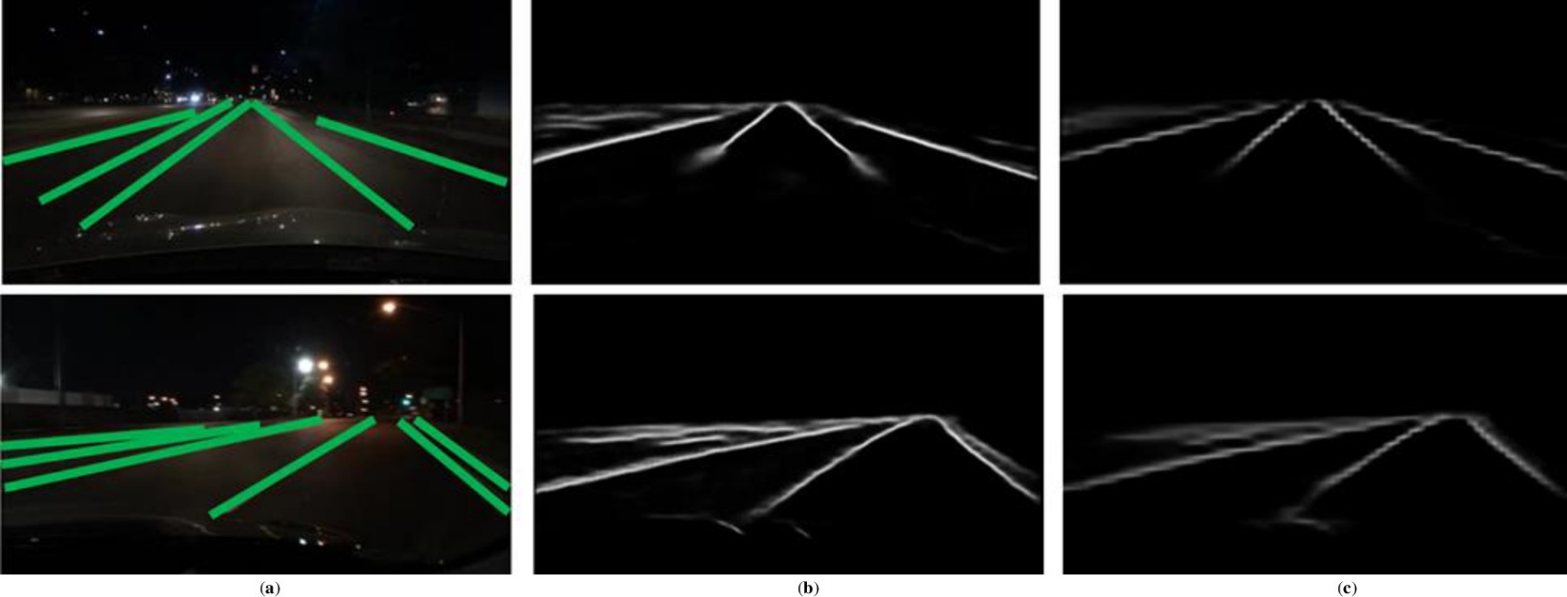
The results of GAAN and SCNN in BDD100K: (**a**) Input and GT; (**b**) GAAN; (**c**) SCNN.

As shown in Fig 10, it displays the lane detection results of GAAN and SCNN on the BDD100K dataset. We selected night scenarios that lane lines are not visible in the testing set. Moreover, BDD100K dataset requires detected lane lines are relatively dense, thus it is more challenging to accurately distinguish the nearby lane lines. From the visualized probabilistic graph of detected lane, we can see that GAAN missed fewer pixels of the lane lines, and the detection results of the lane line which near the driving lane line is better.

## Discussion

In this paper, we have proposed GAAN, a multi-task branches architecture neural network to further improve the ability of lane detection in complex scenes. The one called geometric distance embedding branch can learn the distance features from lane lines’ center to boundary, and the other one called semantic segmentation branch can learn multi-scale semantic features. We use the AIP to adaptively select the complementary information between the two branches for communication and use GAA module to combine the two branches. Consequently, the SPFU is used to fuse the multi-scale features of each stage’s encoder. Experiments were conducted on the CULane dataset, TuSimple dataset, BDD100K dataset and the results show that our method has the better performance compared with several advanced lane detection methods.

In addition, lane detection is an indispensable part of autonomous driving, so it has high requirements on the real-time performance and accuracy of the algorithm, as well as, it needs to control the amount of model parameters to be deployed on the device. Therefore, the further research in future, we must consider that the model of the lane detection method requires real-time detection, and use model compression related technology or a lightweight backbone network to reduce model parameters.

## References

[1] Srivastava S.; Lumb M.; Singal R. Improved lane detection using hybrid median filter and modified hough transform, Int. J. Adv. Res. Comput. Sci. Softw. Eng. 4(1) (2014) 30–37

[2] Narote S.P.; Bhujbal P.N.; Narote A.S. A review of recent advances in lane detection and departure warning system[J]. Pattern Recognition, 2018, 73: 216–234.

[3] Li X.; Li J.; Hu X. Line-CNN: End-to-End Traffic Line Detection With Line Proposal Unit[J]. IEEE Transactions on Intelligent Transportation Systems, 2019, 21(1): 248–258.

[4] Hou.Y.; Ma.Z.; Liu.C. Learning lightweight lane detection cnns by self attention distillation[C]//Proceedings of the IEEE International Conference on Computer Vision. 2019: 1013–1021.

[5] Garnett.N.; Cohen.R.; Pe’er.T. 3D-LaneNet: End-to-end 3D multiple lane detection[C]//Proceedings of the IEEE International Conference on Computer Vision. 2019: 2921–2930.

[6] Amayo.P.; Bruls.T.; Newman.P. Semantic Classification of Road Markings from Geometric Primitives[C]//2018 21st International Conference on Intelligent Transportation Systems (ITSC). IEEE, 2018: 387–393.

[7] Kim J, Park C. End-to-end ego lane estimation based on sequential transfer learning for self-driving cars[C]//Proceedings of the IEEE Conference on Computer Vision and Pattern Recognition Workshops. 2017: 30–38.

[8] Pan.X.; Shi.J.; Luo.P. Spatial as deep: Spatial cnn for traffic scene understanding [C]// Thirty-Second AAAI Conference on Artificial Intelligence. 2018.

[9] Zeng Q, Wang X, Wen H, et al. An empirical investigation of the factors contributing to local-vehicle and non-local-vehicle crashes on freeway[J]. Journal of Transportation Safety & Security, 2020: 1–15. doi: 10.4271/2016-01-1439 27648455 PMC5026383

[10] Wen H, Zhang X, Zeng Q, et al. Bayesian spatial-temporal model for the main and interaction effects of roadway and weather characteristics on freeway crash incidence[J]. Accident Analysis & Prevention, 2019, 132: 105249. doi: 10.1016/j.aap.2019.07.025 31415995

[11] Huang H, Song B, Xu P, et al. Macro and micro models for zonal crash prediction with application in hot zones identification[J]. Journal of transport geography, 2016, 54: 248–256.

[12] Zhang.J.; Xu.Y.; Ni.B. Geometric Constrained Joint Lane Segmentation and Lane Boundary Detection[C]//Proceedings of the European Conference on Computer Vision (ECCV). 2018: 486–502.

[13] Chen.L.C.; Zhu.Y.; Papandreou.G. Encoder-decoder with atrous separable convolution for semantic image segmentation[C]//Proceedings of the European Conference on Computer Vision (ECCV). 2018: 801–818.

[14] He.K.; Zhang.X.; Ren.S. Deep residual learning for image recognition[C]//Proceedings of the IEEE conference on computer vision and pattern recognition. 2016: 770–778.

[15] Huang G.; Liu Z. Densely Connected Convolutional Networks[C] //CVPR. 2017, 1(2): 3.

[16] Audebert N.; Boulch A.; Le.Saux B. Distance transform regression for spatially-aware deep semantic segmentation[J]. Computer Vision and Image Understanding, 2019, 189: 102809.

[17] Felzenszwalb P.F.; Huttenlocher D.P. Distance transforms of sampled functions[J]. Theory of computing, 2012, 8(1): 415–428.

[18] Hayder.Z.; He.X.; Salzmann.M. Boundary-aware instance segmentation[C]//Proceedings of the IEEE Conference on Computer Vision and Pattern Recognition. 2017: 5696–5704.

[19] Bischke.B.; Helber.P.; Folz.J. Multi-task learning for segmentation of building footprints with deep neural networks[C]//2019 IEEE International Conference on Image Processing (ICIP). IEEE, 2019: 1480–1484.

[20] Ghafoorian M, Nugteren C, Baka N, et al. El-gan: Embedding loss driven generative adversarial networks for lane detection[C]//Proceedings of the European Conference on Computer Vision (ECCV) Workshops. 2018: 0–0.

[21] Jifeng Dai, Haozhi Qi, Yuwen Xiong, Yi Li, Guodong Zhang, Han Hu, et al. Deformable convolutional networks. In Proc. IEEE Int. Conf. Comp. Vis., 2017

